# Activities of acyl-CoA:diacylglycerol acyltransferase (DGAT) and phospholipid:diacylglycerol acyltransferase (PDAT) in microsomal preparations of developing sunflower and safflower seeds

**DOI:** 10.1007/s00425-013-1870-8

**Published:** 2013-03-29

**Authors:** Walentyna Banaś, Alicia Sanchez Garcia, Antoni Banaś, Sten Stymne

**Affiliations:** 1 Institute of Biology, University of Natural Sciences and Humanities, Prusa 12, 08-110 Siedlce, Poland; 2 Instituto de la Grasa, CSIC, Sevilla, Spain; 3 Intercollegiate Faculty of Biotechnology, University of Gdansk and Medical University of Gdansk, Kladki 24, 80-822 Gdansk, Poland; 4 Department of Plant Breeding, SLU, Alnarp, Sweden

**Keywords:** PDAT, DGAT, Sunflower, Safflower, Microsomal preparation, Triacylglycerols, Lipids

## Abstract

The last step in triacylglycerols (TAG) biosynthesis in oil seeds, the acylation of diacylglycerols (DAG), is catalysed by two types of enzymes: the acyl-CoA:diacylglycerol acyltransferase (DGAT) and phospholipid:diacylglycerol acyltransferase (PDAT). The relative contribution of these enzymes in the synthesis of TAG has not yet been defined in any plant tissue. In the presented work, microsomal preparations were obtained from sunflower and safflower seeds at different stages of development and used in DGAT and PDAT enzyme assays. The ratio between PDAT and DGAT activity differed dramatically between the two different species. DGAT activities were measured with two different acyl acceptors and assay methods using two different acyl-CoAs, and in all cases the ratio of PDAT to DGAT activity was significantly higher in safflower than sunflower. The sunflower DGAT, measured by both methods, showed significant higher activity with 18:2-CoA than with 18:1-CoA, whereas the opposite specificity was seen with the safflower enzyme. The specificities of PDAT on the other hand, were similar in both species with 18:2-phosphatidylcholine being a better acyl donor than 18:1-PC and with acyl groups at the *sn*-2 position utilised about fourfold the rate of the *sn*-1 position. No DAG:DAG transacylase activity could be detected in the microsomal preparations.

**Electronic supplementary material:**

The online version of this article (doi:10.1007/s00425-013-1870-8) contains supplementary material, which is available to authorized users.

## Introduction

Triacylglycerols (TAG) are the main components of seeds’ storage lipids in oil seed plants, (Stymne and Stobart 1987; Murphy 2005). TAG biosynthesis in seed cells is thought to occur in endoplasmic reticulum (ER), and TAG accumulates in oil bodies generated through budding off from the outer ER membrane (Stymne and Stobart 1987; Huang 1992; Somerville et al. 2001; Chapman et al. 2012). TAG can be synthesised by enzymes of Kennedy pathway (Kennedy 1961) via sequential acylation of the glycerol backbone with three *sn*-specific acyltransferases, transferring acyl chains from acyl-CoA (Ohlrogge and Browse 1995). Until rather recently, it was assumed that the last step in TAG biosynthesis is uniquely catalysed by acyl-CoA:diacylglycerol acyltransferase (DGAT) acylating the *sn*-3 position of *sn*-1, 2-DAG. The DGAT genes were first cloned from mouse (Cases et al. 1998) and then from plants (Hobbs et al. 1999; Zou et al. 1999; Bouvier-Nave et al. 2000). It was later discovered that many organism have two distinct classes of DGATs with no homology to each other (DGAT1 and DGAT2), (Lardizabal et al. 2001; Kroon et al. 2006; Shockey et al. 2006; Zhang et al. 2009; Liu et al. 2012). Additionally, a soluble form of DGAT was recently cloned (Saha et al. 2006; Hernandez et al. 2012). In addition to DGAT-catalysed TAG formation, it was shown that an acyl-CoA-independent biosynthesis of TAG exists in yeast and plants. The enzyme involved in this process, the phospholipid:diacylglycerol acyltransferase (PDAT), transfers an acyl group from the *sn*-2 position of phospholipids (e.g. phosphatidylcholine, phosphatidylethanolamine) to *sn*-3 position of diacylglycerol, yielding TAG and *sn*-1-lysophospholipid (Banas et al. 2000; Dahlqvist et al. 2000; Oelkers et al. 2000; Ståhl et al. 2004). It has been shown from studies of mutants and down regulation by RNAi that PDAT1 and DGAT1 are the two main enzymes contributing to seed TAG synthesis in Arabidopsis (Zhang et al. 2009). However, the relative contribution of both types of enzymes in TAG biosynthesis cannot be determined by these studies. A mutation in PDAT gene could, e.g. be fully compensated in TAG synthesis by the remaining DGAT (Mhaske et al. 2005). In the presented study, we measured the DGAT and PDAT activity in the microsomal fractions from developing seeds of sunflower (*Helianthus annuus* L.) and safflower (*Carthamus tinctorius* L.) and show that the relative contribution of the two types of enzymes in TAG synthesis differed dramatically in those two species.

## Materials and methods

### Plant material

Seeds of sunflower (*H. annuus* L.; line HA89; a medium-high oleic line) and safflower (*C. tinctorius* L., cv. Gila, a very high linoleic variety) were planted on peat-based soil and transferred to the growth chamber with 70 % relative humidity and 16-h photoperiod (200 μmol radiation; day temperature of 25 °C; night temperature of 20 °C). After a few weeks, the plants started flowering. Sunflower is a self-pollinating plant, while the safflower needed to be pollinated manually. At certain days after beginning of flowering (DAF) or after pollination (DAP), the developing seeds were harvested manually and used for microsomal preparations and for lipid analyses.

### Substrates

Radioactive fatty acids and di-[1-^14^C]18:1-PC, were obtained from Perkin Elmer. *Sn*-1-18:1-lyso-PC, *sn*-1-monoacylglycerols, di-18:1-DAG, di-6:0-DAG and unlabelled fatty acids were purchased from Sigma. *Sn*-1-18:1-lyso-PE and *sn*-1-[^14^C]18:1-lyso-PC were obtained by phospholipase (*Naja mossambica mossambica*; Sigma) treatment of di-18:1-PE (Avanti Polar Lipids, Birmingham, AL) and di-[1-^14^C]18:1-PC, respectively, followed by purification by TLC. The synthesis of PC, and PE with ^14^C-labelled acyl groups at position *sn*-2 were done by chemical acylation of the corresponding [^14^C]acyl tri-fluoro anhydride to 18:1-lyso-PC and 18:1-lyso-PE (Kanda and Wells 1986). *Sn*-1-[^14^C]18:1-*sn*-2-18:1-PC was synthesised similarly by chemical acylation of *sn*-1-[^14^C]18:1-lyso-PC with the tri-fluoro anhydride derivative of 18:1. Radioactive DAG (*sn*-1-18:1-*sn*-2-[^14^C]18:1-DAG and di-6:0-[^14^C]DAG) was synthesised by reacting monoacylglycerol or [^14^C]glycerol (in case of radioactive di-6:0-DAG) with the tri-fluoro anhydride of the radioactive or non-radioactive fatty acids. Acyl-vernoleoyl-DAG was prepared from *Crepis palaestina* TAGs by partial lipase treatment (*Rizhopus arrhizus*; Sigma). All lipid products were separated on TLC, eluted from the gel and concentration determined by analysing the fatty acid content of aliquots as methyl esters on GLC with methyl-heptadecanoic acid added as an internal standard as described below.

### Microsomal preparation and enzyme assays

Microsomal membranes were prepared from freshly harvested seeds (at different stages of development). The seeds coats were removed manually and microsomes were prepared according to the method previously described (Stobart and Stymne 1985) and stored at −80 °C until used for assays. DGAT activity was measured in assays with two different acceptors of fatty acids: di-6:0-DAG (only DGAT assays) and *sn*-1-18:1-*sn*-2-[^14^C]18:1-DAG (DGAT + PDAT and PDAT assays). In assays with di-6:0-DAG, 5 nmol [^14^C]18:1-CoA or 5 nmol [^14^C]18:2-CoA together with 5 nmol di-6:0-DAG were added to the microsomal membranes (26 μg of microsomal protein, which was equivalent to approximately 6 nmol of microsomal phosphatidylcholine for both species) with incubation buffer (0.05 M HEPES–pH 7.2; 5 mM MgCl_2_; 1 mg BSA/ml) in a final volume 100 μl and incubated 5 min at 30 °C with shaking (1,250 rpm). In case of DGAT + PDAT and PDAT assays, 5 nmol [^14^C]18:1-DAG was dissolved in 19 μl of benzene and added to overnight lyophilised aliquots of microsomal fractions (corresponding to 26 μg of microsomal protein). After immediate evaporation of the solvent, buffer (0.05 M HEPES–pH 7.2; 5 mM MgCl_2_, 1 mg BSA/ml) was added and, in case of measuring combined DGAT + PDAT activities, additionally 5 nmol acyl-CoA. The assays (final volume 100 μl) were incubated for 5 min at 30 °C with shaking (1,250 rpm). In assays with [^14^C]18:1-DAG + acyl-CoA, formation of [^14^C]TAG was regarded as a result of both DGAT and PDAT activity. Formation of [^14^C]TAG in assays with only [^14^C]18:1-DAG added was regarded as only PDAT activity. DGAT activity was calculated as amount of [^14^C]TAG in assays with [^14^C]DAG + acyl-CoA minus the amount of [^14^C]TAG in assays with [^14^C]DAG only. Linearity was not observed at any time point, but all assays showed the same increase of 100 % in product formation between 5 min and 15 min incubation (Supplement tables S1, S2, S3 and S4). In assays determining PDAT’s acyl donor specificities, unlabelled di-18:1-DAG (5 nmol) and 5 nmol *sn*-2[^14^C]acyl-PC, *sn*-1[^14^C]18:1-PC or *sn*-2[^14^C]acyl-PE dissolved in 19 μl benzene were added to the freeze-dried microsomes (corresponding to 26 μg microsomal protein) and solvent was immediately evaporated. Other incubation conditions were the same as described above.

At the end of incubation, lipids were extracted from reaction mixtures into chloroform according to Blight and Dyer (1959) and separated on TLC (silica gel 60 plates; Merck, Darmstadt, Germany) in hexane:diethyl ether:acetic acid (70:30:1 by volume). Radioactive TAG (TAG with two 6:0 moieties clearly separated on TLC from TAG with only long chain fatty acids), products of PDAT and DGAT activity, were visualised and quantified on the plate using electronic autoradiography (Instant Imager, Packard instruments).

All assays were repeated at least three times and mean values with standard deviations are presented in the tables.

### Lipid analysis

Seeds were homogenised in chloroform:methanol:0.15 M acetic acid (1:2:0.8) using a Potter–Elvehjem homogenizer and the lipids were subsequently extracted into chloroform according to Blight and Dyer (1959). For total lipids analysis, aliquots of the chloroform phase was evaporated and methylated as described below. Individual lipids in the chloroform phase were separated by TLC in hexane:diethyl ether:acetic acid (70:30:1) for neutral lipids or in chloroform:methanol:acetic acid:water (85:15:10:3.5) for separation of polar lipids. Gel from areas corresponding to analysed lipid classes (identified by mean of standards), was removed and lipids were methylated in situ on the gel with 2 % H_2_SO_4_ in dry methanol (60 min at 90 °C). The methyl esters were extracted with hexane and analysed by GLC equipped with a flame ionisation detector and a WCOT fused-silica 50 m × 0.32 mm ID coating CP-Wax 58-CB DF 5 0.2 capillary column (Chrompack International, Middleburg, The Netherlands) with methyl-heptadecanoic acid added as an internal standard.

## Results

### Lipid accumulation in sunflower and safflower seeds

**Fig. 1:**
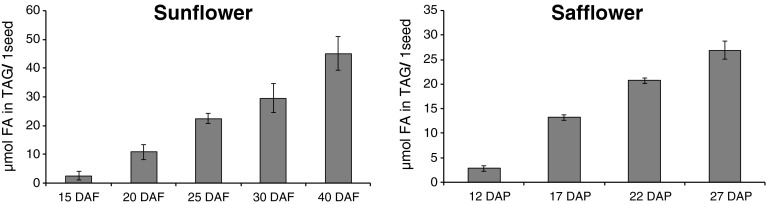
Accumulation of triacylglycerols in developing sunflowers and safflower seeds. *DAF* days after flowering, *DAP* days after pollination

The first samples for lipid content and composition from developing sunflower seeds begun at 15 days after flowering (DAF) and in case of safflower seeds at 12 days after pollination (DAP). At these time points of seeds development, the lipid accumulation in the seeds was rather small and ranged from around 6 (sunflower) to 10 % (safflower) of the final amount of lipids of mature seeds. In later stages of seeds development, the increase in lipid content per seed was almost linear until the end of the experiment (40 DAF in case of sunflower and 27 DAP in case of safflower), (Fig. 1). During that time the seeds almost reached their maturity and the amount of the lipid content was similar to the dry seeds (data not shown).

The dominant lipid classes in both sunflower and safflower seeds, in all analysed stages of seeds development, were TAG (Tables 1, 2). In case of sunflower, its relative amount ranged between around 90 % (15 DAF) and 97 % (40 DAF) and in case of safflower from about 87 % (12 DAP) to 97 % (27 DAP). The amount of polar lipids was relatively high at the beginning of seed development (about 9–11 % of total lipids in sunflower and safflower seeds, respectively), but accounted only for about 1.3 % (sunflower) to 1.5 % (safflower) of total lipid amount in the mature seeds. Relative amounts of DAG in the developing sunflower seeds ranged from 0.7 to 1.3 % of the total lipids and from about 0.9 to 1.4 % in developing safflower seeds. The amount of free fatty acids and sterol esters was low and did not exceed 0.3 % of total fatty acid content of seed lipids in both analysed plant species (Tables 1, 2).

**Table 1:** Distribution of lipid classes in developing sunflower seeds

Stage of development	% of total fatty acids				
	Polar lipids	DAG	FA	TAG	SE
15 DAF	8.3 ± 0.8	0.8 ± 0.1	0.29 ± 0.01	89.9 ± 0.7	0.3 ± 0.1
20 DAF	4.6 ± 0.3	0.9 ± 0.1	0.09 ± 0.04	93.3 ± 1.0	0.9 ± 0.5
25 DAF	3.1 ± 0.1	1.3 ± 0.1	0.12 ± 0.01	94.9 ± 0.5	0.6 ± 0.3
30 DAF	2.1 ± 0.1	1.4 ± 0.1	0.09 ± 0.02	95.9 ± 0.1	0.5 ± 0.2
40 DAF	1.3 ± 0.1	0.7 ± 0.1	0.06 ± 0.02	97.9 ± 0.3	0.4 ± 0.2

Mean ± SD shown (*n* ≥ 3)

*DAF* days after flowering, *DAG* diacylglycerols, *FA* free fatty acids, *TAG* triacylglycerols, *SE* sterol esters

**Table 2:** Distribution of lipid classes in developing safflower seeds

Stage of development	% of total fatty acids				
	Polar lipids	DAG	FA	TAG	SE
12 DAP	10.5 ± 0.5	1.4 ± 0.1	0.25 ± 0.05	86.9 ± 0.1	0.4 ± 0.1
17 DAP	3.6 ± 0.1	0.9 ± 0.1	0.15 ± 0.05	95.2 ± 0.1	0.2 ± 0.1
22 DAP	2.5 ± 0.4	1.1 ± 0.1	0.15 ± 0.05	96.5 ± 0.1	0.2 ± 0.1
27 DAP	1.6 ± 0.2	0.9 ± 0.1	0.10 ± 0.01	97.3 ± 0.2	0.2 ± 0.1

Mean ± SD shown (*n* ≥ 3)

*DAP* days after pollination, *DAG* diacylglycerols, *FA* free fatty acids, *TAG* triacylglycerols, *SE* sterol esters

Oleic acid was the dominating fatty acid in TAG from developing sunflower seeds. Its relative amount increased from 63 % at 15 DAF to 68 % at 20 DAF (Table 3). Between 20 and 25 DAF, its relative amounts decreased only slightly and after that the decrease was more significant. At 40 DAF, it amounted to 59 % of TAG fatty acids. Linoleic acid was the second most abundant among TAG fatty acids with fluctuations showing opposite trends to oleic acid. Its relative amount at 15 DAF was 20 %, after which it decreased to about 17 % at 20 DAF and then increased up to about 32 % at 40 DAF. Palmitic and stearic acid amounts ranged from about 10 % to about 4 % of total TAG’s fatty acids. All other TAG’s fatty acids were detected in only trace amounts.

**Table 3:** Fatty acids composition of triacylglycerols from developing sunflower seeds

Stage of development	FA (mol %)				
	16:0	18:0	18:1	18:2	18:3
15 DAF	7.3 ± 0.3	9.7 ± 0.5	62.8 ± 1.2	19.9 ± 1.9	0.3 ± 0.1
20 DAF	6.6 ± 0.2	7.2 ± 0.1	68.8 ± 0.2	17.4 ± 0.3	0.1 ± 0.1
25 DAF	3.9 ± 0.1	6.7 ± 0.1	68.1 ± 0.6	20.6 ± 0.2	0.1 ± 0.1
30 DAF	4.6 ± 0.4	5.5 ± 0.5	65.5 ± 1.5	24.6 ± 1.3	0.1 ± 0.1
40 DAF	5.1 ± 0.2	4.2 ± 0.1	59.0 ± 0.2	31.5 ± 1.2	0.1 ± 0.1

Mean ± SD shown (*n* ≥ 3)

*DAF* days after flowering

Linoleic acid accounted for about 88–91 % of total fatty acids in TAG of developing safflower seeds (Table 4). The relative amounts of oleic and palmitic acids ranged from about 6 to about 3 % and the relative amount of stearic acid was about 1 % of total TAG’s fatty acids.

**Table 4:** Fatty acids composition of triacylglycerols from developing safflower seeds

Stage of development	FA (mol %)				
	16:0	18:0	18:1	18:2	18:3
12 DAP	4.3 ± 0.2	1.2 ± 0.1	5.9 ± 0.2	87.8 ± 1.2	0.3 ± 0.2
17 DAP	3.8 ± 0.1	0.8 ± 0.1	4.0 ± 0.1	91.0 ± 0.2	0.3 ± 0.1
22 DAP	2.9 ± 0.1	0.9 ± 0.1	4.5 ± 0.1	91.2 ± 0.1	0.3 ± 0.1
27 DAP	3.4 ± 0.1	1.0 ± 0.1	4.3 ± 0.1	91.0 ± 0.1	0.2 ± 0.1

Mean ± SD shown (*n* ≥ 3)

*DAP* days after pollination

The fatty acids composition of polar lipid classes and DAG of both sunflower and safflower developing seeds reflected the fatty acids composition of TAG as described above. The biggest difference was that the relative amount of palmitic acids was somewhat higher than in TAG (results not shown).

### PDAT and DGAT activities and their substrate specificities in developing sunflower and safflower seeds

PDAT and DGAT activities were measured in microsomal fractions prepared from developing sunflower and safflower seeds. The seeds used for preparation of microsomal fractions were collected at the same time as seeds collected for lipid analyses (see above). The rate of synthesised [^14^C]TAG from exogenous [^14^C]18:1-DAG and endogenous phospholipids was regarded as a result of PDAT activity. DGAT activity was calculated as difference between the amount of synthesised [^14^C]TAG from exogenous [^14^C]18:1-DAG + acyl-CoA (PDAT + DGAT activity) minus average value of PDAT activity. We also measured DGAT activity using endogenous DAG by adding radioactive acyl-CoA. The activity was lower than measured with above-described methods (Supplement Table S5, Table S6). If we had added non-radioactive long chain DAG together with radioactive acyl-CoA, we might have had higher activities but we would not have control over how much of the added versus endogenous DAG was used by the DGAT and how that related to the pool of DAG that was used by PDAT. In this context, it should be mentioned that in vivo labelling studies of developing oil seeds indicate that there are at least two pools of DAG in oil seeds; one that comes directly via glycerol 3-phosphate pathway and one that is derived from PC and utilised in TAG synthesis (Bates and Browse 2012). It is not known if such putative separate DAG pools are maintained in our microsomal fraction and how the introduced DAG substrate in such case is partitioned between the pools. By measuring both DGAT and PDAT on the same pool of radioactive DAG, we avoid the possibilities that the assay results are based on utilisation of different pools or pool sizes of acyl acceptor by the two different enzymes.

For DGAT assays, two different DAG molecules were used: [^14^C]18:1-DAG and di-6:0-DAG (non-radioactive). Incubations with [^14^C]di-6:0-DAG in absence of acyl-CoA produced only trace amounts of [^14^C]di-6:0-TAG, whereas radioactive di-6:0-TAG was efficiently produced by addition of unlabelled 18:1-CoA to the same incubations (Supplement Fig. 1). These experiments demonstrate that PDAT, contrary to DGAT, could not utilise di-6:0-DAG as acyl acceptor and that the microsomal preparations only contained trace amounts of endogenous acyl-CoA. Thus, in contrast to the assays with di-18:1-DAG, assays with di-6:0-DAG as an acyl acceptor measured only DGAT activity. In case of experiments with non-radioactive di-6:0-DAG and radioactive acyl-CoA, only small amount radioactive TAG was formed from endogenous DAG compared to di-6:0-TAG (in case of sunflower it ranged from 0.4 to 4.8 % and in case of safflower 2.3–14.3 % of total radioactivity in TAG, data not shown).

In developing seeds of sunflower, DGAT activity was dominating over the PDAT activity in all stages of seeds development when measured both with 18:1-DAG and 6:0-DAG as acyl acceptors (Table 5). In contrast to sunflower microsomes, PDAT activity dominated over DGAT activity in microsomal membranes prepared from most stages of safflower seeds when 18:1-DAG was used as acyl acceptor (Table 6). Since DGAT assays with 6:0-DAG gave significantly higher amount of de novo-produced TAG compared to assays with 18:1-DAG, the DGAT activity measured with this acyl acceptor always gave higher activity than the PDAT activity. The most probable explanation for the higher DGAT activity with 6:0-DAG than with 18:1-DAG is that the 6:0-DAG is more accessible to the enzyme than introduced long chain DAG but also endogenous DAG, since 6:0-DAG virtually outcompeted the acylation of endogenous DAG. It could also be due to extremely much higher specificity for 6:0-DAG than for long chain DAG, but this is more unlikely since such short chain DAG does not exist in those seeds.

**Table 5:** TAG synthesis from different combinations of substrates by microsomal fractions from developing sunflowers seeds

Substrates added	nmol [^14^C]TAG formed/min × mg protein				
	15 DAF	20 DAF	25 DAF	30 DAF	40 DAF
[^14^C]18:1DAG (PDAT activity)	0.19 ± 0.05	0.25 ± 0.06	0.22 ± 0.04	0.22 ± 0.04	0.22 ± 0.05
[^14^C]18:1-DAG + 18:1-CoA (PDAT + DGAT activity)	1.72 ± 0.22	1.78 ± 0.28	1.59 ± 0.24	1.37 ± 0.22	1.34 ± 0.08
Calculated DGAT activity	1.53	1.53	1.37	1.15	1.12
[^14^C]18:1DAG + 18:2-CoA (PDAT + DGAT activity)	2.93 ± 0.17	4.16 ± 0.28	3.90 ± 0.26	3.58 ± 0.17	2.32 ± 0.06
Calculated DGAT activity	2.74	3.91	3.68	3.36	2.1
di-6:0-DAG + [^14^C]18:1-CoA (DGAT activity)	2.82 ± 0.86	3.00 ± 0.21	1.88 ± 0.12	1.82 ± 0.04	1.79 ± 0.19
di-6:0-DAG + [^14^C]18:2-CoA (DAGAT activity)	3.18 ± 0.12	6.48 ± 0.18	3.97 ± 0.14	3.80 ± 0.43	3.22 ± 0.08

In experiments with di-6:0-DAG + [^14^C]FA-CoA only synthesised di-6:0-TAG is presented; the amount of [^14^C]TAG synthesised from endogenous DAG and [^14^C]FA-CoA was low (from 0.7 to 4.8 % of synthesised di-6:0-TAG value). Mean ± SD shown (*n* ≥ 3)

*DAF* seeds days after flowering used for microsomal membrane preparations

**Table 6:** TAG synthesis from different combinations of substrates by microsomal fractions from developing safflower seeds

Substrates added	nmol [^14^C]TAG formed/min × mg protein			
	12 DAP	17 DAP	22 DAP	27 DAP
[^14^C]18:1-DAG (PDAT activity)	0.57 ± 0.08	0.66 ± 0.09	0.68 ± 0.04	0.36 ± 0.02
[^14^C]18:1-DAG + 18:1-CoA (PDAT + DGAT activity)	1.56 ± 0.27	1.16 ± 0.04	1.16 ± 0.12	0.60 ± 0.03
Calculated DGAT activity	0.99	0.50	0.48	0.24
[^14^C]-18:1DAG + 18:2-CoA (PDAT + DGAT activity)	0.99 ± 0.08	0.92 ± 0.09	0.89 ± 0.04	0.51 ± 0.08
Calculated DGAT activity	0.42	0.26	0.21	0.15
di-6:0-DAG + [^14^C]18:1-CoA (DGAT activity)	3.28 ± 0.26	2.57 ± 0.08	2.10 ± 0.17	1.67 ± 0.06
di-6:0-DAG + [^14^C]18:2-CoA (DGAT activity)	2.02 ± 0.13	1.98 ± 0.05	1.69 ± 0.06	0.95 ± 0.1

In experiments with di-6:0-DAG + [^14^C]FA-CoA only synthesised di-6:0-TAG is presented; the amount of [^14^C]TAG synthesised from endogenous DAG and [^14^C]FA-CoA was low (from 2.3 to 14.3 % of synthesised di-6:0-TAG value). Mean ± SD shown (*n* ≥ 3)

*DAP* seeds days after pollination used for microsomal membrane preparations

Sunflower microsomal DGAT utilised 18:2-CoA better than 18:1-CoA. Its activity was in most assays more than twice that of 18:1-CoA (Table 5). In assays with safflower microsomes, 18:1-CoA was better utilised by DGAT of microsomal fractions than 18:2-CoA. These preferences were not dependent on the DAG molecules (di-18:1-DAG or di-6:0-DAG) and were observed in assays with microsomal fraction prepared at all stages of seeds development (Tables 5, 6).

The specific DGAT activity in safflower membranes was highest in young seeds (12 DAP) and then gradually decreased. In case of microsomal membranes from sunflower seeds, the highest DGAT activity was observed at 20 DAF and then decreased gradually reaching 50–70 % of its highest value at 40 DAF (Tables 5, 6).

**Fig. 2:**
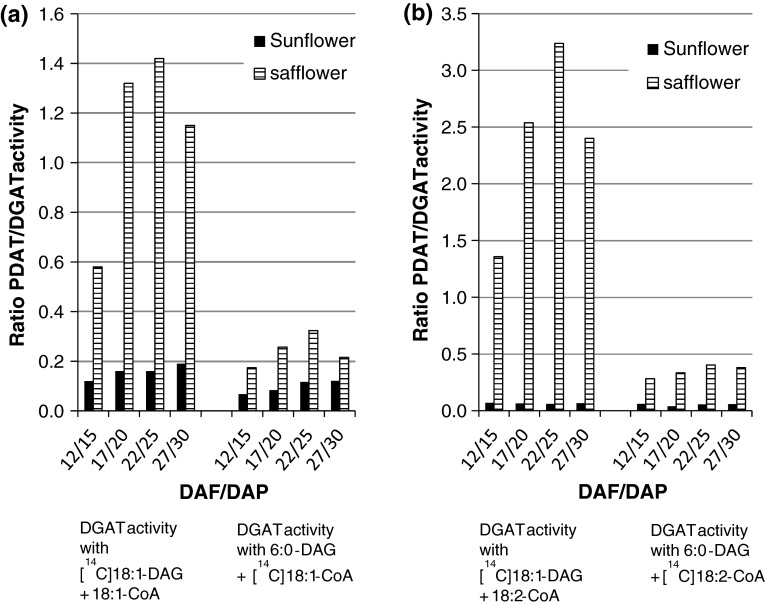
Ratios of PDAT/DGAT activities in microsomal preparations from developing sunflower and safflower seeds at different days after flowering (DAF) or pollination (DAP), respectively. DGAT activity was measured with two different molecular species of DAG and two different acyl-CoAs: **a** 18:1 CoA, **b** 18:2 CoA. The data are calculated from values given in Tables 5, 6

The ratios between PDAT and DGAT activities in sunflower and safflower membranes at different stages of development using di-18-DAG, di-6:0 DAG, 18:1-CoA or 18:2-CoA as DGAT substrates are depicted graphically in Fig. 2. In all cases, the ratios were significantly higher in safflower membranes than in sunflower membranes.

In substrate specificity studies of microsomal PDAT, combination of di-18:1-DAG and four different radioactive molecular species of PC and two different molecular species of PE (fatty acid donors) were used. Except for one PC substrate, all tested phospholipid substrates had ^14^C-labelled fatty acids in position *sn*-2 (PC: 18:0, 18:1, 18:2; PE: 18:1, 18:2) and non-radioactive 18:1 in position *sn*-1. A PC species with the radioactive 18:1 at *sn*-1 position and unlabelled 18:1 at *sn*-2 position was also included in the study. The PDAT activity was measured as the amount of synthesised [^14^C]TAG. From the tested combination of exogenous DAG and ^14^C-labelled phospholipids, PE with 18:2 was the best acyl donor for sunflower PDAT, followed by PE with 18:1, PC with 18:2, PC with 18:1 and PC with 18:0 at *sn*-2 position. Similar tendency was also observed for safflower PDAT with one exception; PC with 18:2 was better utilised than PE with 18:1. PDAT of both tested species utilised 18:1 from position *sn*-1 of PC at about 25 % of the rate compared to position *sn*-2 (Table 7).

**Table 7:** Substrate specificity of PDAT in microsomal preparations from developing (middle-stage) sunflower and safflower seeds

pmol [^14^C]TAG formed/min × mg protein					
Substrates: 5 nmol *sn*1, *sn*2-di-18:1-DAG + 5 nmol of [^14^C]phospholipid indicated below					
PC; *sn*1-18:1- *sn*2-[^14^C]18:0	PC; *sn*1-18:1- *sn*2-[^14^C]18:1	PC; *sn*1-18:1- *sn*2-[^14^C]18:2	PC; *sn*1-[^14^C]18:1-*sn*2-18:1	PE; *sn*1-18:1- *sn*2-[^14^C]18:1	PE; *sn*1-18:1- *sn*2-[^14^C]18:2
Plant					
Sunflower					
23 ± 3 (55)	42 ± 3 (100)	75 ± 8 (179)	10 ± 2 (24)	85 ± 2 (202)	163 ± 13 (388)
Safflower					
27 ± 2 (47)	57 ± 12 (100)	205 ± 8 (360)	12 ± 3 (21)	122 ± 15 (214)	417 ± 75 (732)

Mean ± SD shown (*n* ≥ 3); figures in parentheses show relative PDAT activity (activity towards *sn*1-18:1-*sn*2-[^14^C]18:1-PC was treated as 100 %)

In order to investigate whether the microsomal fractions of sunflower and safflower seeds possess DAG:DAG acyltransferase activity, a mixture of *sn*-1-18:1-*sn*-2-[^14^C]18:1-DAG and *rac*-*sn*-1-non-epoxy-acyl-*sn*-2-vernoleoyl-DAG were used as substrates employing the same incubation conditions as in the PDAT assays. Transfer of [^14^C]18:1 to the vernoleoyl-containing DAG or transfer of vernoleoyl groups to the radioactive di-18:1-DAG would be detected as radioactive TAG containing one epoxy fatty acid, which have different mobility than TAG with no epoxy groups on TLC. With these substrate combinations, no traces of [^14^C]-1-epoxy-TAG were detected. Only [^14^C]TAG with common fatty acids was synthesised, catalysed by PDAT. If the TLC plates were stained with I_2_, the non-radioactive 1-epoxy-TAG was clearly visible, presumably also formed by PDAT. Thus, the experiment failed to show any DAG:DAG acyltransferase activity (results not shown).

## Discussion

**Scheme 1:**
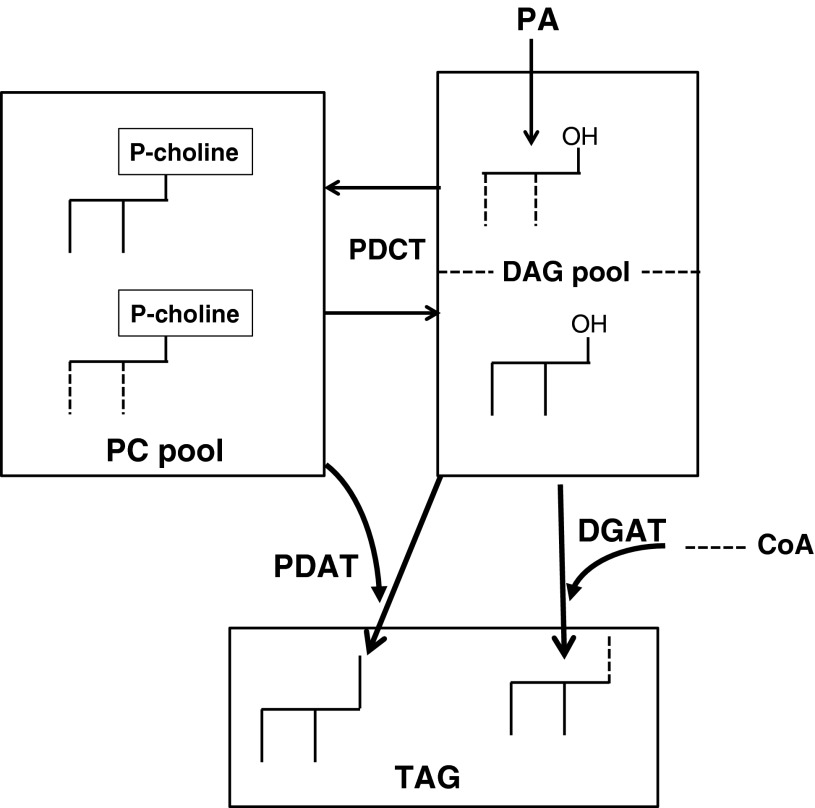
Schematic model of substrate pools used by PDAT and DGAT enzymes in oil seeds with high DAG-PC equilibration activities. *Dotted lines* are acyl groups derived from acyl-CoA pool and *filled lines* are acyl groups derived from phosphatidylcholine (PC). Phosphatidic acid (PA) derived from glycerol 3-phosphate pathway are converted to a diacylglycerol (DAG) that is interconverted with PC in a reaction catalysed by phospholipid:diacylglycerol cholinephosphotransferase (PDCT) and possibly also CDP-choline:diacylglycerol phosphocholine transferase (CPT). PDAT transfer an acyl groups from PC to DAG mainly derived from PC, in formation of triacylglycerols, whereas DGAT transfer an acyl group from acyl-CoA to DAG mainly derived from PC. The model suggests at least two DAG pools and is based on in vivo labelling experiments (Bates and Browse 2012)

Two types of enzymes are involved in the last step of TAG synthesis in oil seeds plants: DGAT and PDAT. The two enzymes utilise both diacylglycerols (DAG) that could either be produced by the Kennedy pathway (Kennedy 1961) or derived from interconversion with PC (Bates and Browse 2012). The generation and utilisation of the DAG pools by PDAT and DGAT is schematically depicted in Scheme 1. The key roles of DGAT and PDAT enzymes in TAG synthesis in *Arabidopsis* seeds have recently been demonstrated in studies of mutants and RNAi inhibition of those genes (Zhang et al. 2009), but their relative contribution to TAG accumulation has not been determined in any species. Silencing PDAT or DGAT encoding genes cannot be used to elucidate the relative contribution of the two enzymes in TAG accumulation since the remaining active enzyme might compensate for much of the loss of the other enzyme (Mhaske et al. 2005; Zhang et al. 2009). One approach to determine the relative contribution of PDAT and DGAT is to determine their relative activities in vitro. However, measurements of these in vitro activities are problematic. In vitro activities of PDAT have only been reported from our laboratory (Banas et al 2000; Dahlqvist et al. 2000; Ståhl et al. 2004). The reports on DGAT activity in microsomal preparation of developing oils seeds show huge variation in specific activity, ranging from 1.5 to 14,500 pmol per min and mg microsomal protein (Wiberg et al. 1994; Sörensen et al. 2005). Similarly, reports of specific activities of DGAT in microsomal preparations of developing safflower and sunflower seeds ranged from 20 to 5,000 pmol/min × mg protein and from 15 to 5,000 pmol/min × mg protein, respectively (Weselake et al. 1993; Vogel and Browse 1996; Triki et al. 2000; Wiberg et al. 1994). It can be assumed that at least some of these discrepancies between different reports, which are based on measuring amount of radioactive substrates incorporated into TAG, can be due to dilution of added substrates with endogenous substrates. Added radioactive DAG will be diluted with endogenous DAG in the membranes and added acyl-CoA could be diluted with endogenous acyl groups arising from acyl exchange with phosphatidylcholine (Stymne and Stobart 1984). Addition of membrane-bound substrates necessitates a method for integrating these substrates into membranes in a way that make them accessible for the enzymes. This is usually done by addition of detergent. Although good activity of DGAT has been obtained by adding DAG in detergents (Wiberg et al. 1994), they severely decrease the activity of PDAT (Stymne et al., unpublished observation). The method used to add the substrate in benzene to freeze-dried microsomes is the only assay method reported to work for membrane-bound PDAT (Banas et al. 2000; Dahlqvist et al. 2000; Ståhl et al. 2004). Since the main aim of the present work was to assess the relative contribution of DGAT and PDAT in TAG synthesis, we therefore measured the DGAT under exactly the same conditions as PDAT, using the same radioactive substrate, i.e. radioactive DAG. Dilution of the radioactive DAG with endogenous DAG or via PC-DAG interconversion (Lu et al. 2009) will thus be the same for both enzyme assays. We did not separate individual polar lipids from the assays, but the radioactivity in the application spot, where all the polar lipids are, was low. In the experiments with sunflower microsomes we detected about 5 % of total added radioactivity in polar lipids, and in the experiments with safflower microsomes about 2 % (data not shown). This small dilution of endogenous PC by [^14^C]PC will have a very negligible effect on measured PDAT activity (measured as conversion of [^14^C]DAG into TAG with endogenous phospholipid as acyl donors) in both sunflower and safflower microsomes. Consequently, also the acyl exchange of acyl group between synthesised [^14^C]PC and added exogenous acyl-CoA will also be marginal, and thus not affected the measured DGAT activity. Comparing our assays of activities of PDAT measured with radioactive PC and non-radioactive DAG as added substrates with added radioactive DAG using endogenous phospholipids as acyl donor illustrates the caveats of comparing assays with different substrates. The assays with radioactive DAG gave about three times higher PDAT activity compared to radioactive PC with 18:2 at position *sn*-2 and five (sunflower) to 11 (safflower) times compared to radioactive PC with 18:1 at position *sn*-2. The proportions of PC and DAG in sunflower and safflower membranes ranged from about 4:1 to 6:1 (data not presented). Based on these pool sizes, substrate dilution can only explain up to about twofold increase in apparent activity using radioactive DAG as substrate compared to radioactive PC.

In the present assays we tried to eliminate variation depending on assay conditions as much as possible by employing the same amount of microsomal protein and other assay conditions for both sunflower and safflower membranes. Further, the incubation time was kept short to minimise the deviation seen from linearity and extent the substrates were used in other reactions that would skew the measured activities, such as by acyl exchange or PC-DAG equilibration. The specific activities achieved for DGAT were in the same magnitude of the highest reported in microsomal membranes from these seeds (Vogel and Browse 1996; Wiberg et al. 1994). The ratio between PDAT and DGAT activity were always much higher in safflower membranes. Measured under the same incubation conditions, the ratios were between 19 and 54 times higher in safflower, depending on developmental stage and acyl-CoA species (Fig. 2). When the ratios were calculated based on DGAT activity with 6:0-DAG, they were 2–7 times higher in safflower compared to sunflower membranes.

In case of sunflower, DGAT preferentially utilised 18:2-CoA compared to 18:1-CoA and in case of safflower it was the opposite, regardless of whether the acyl acceptor was 18:1 DAG or the artificial 6:0-DAG. The main fatty acid of TAG of the tested variety of sunflower is oleic acid, and in safflower TAGs it is linoleic acid. The higher specialisation of sunflower DGAT towards linoleic acid could be explained by the fact that linoleic acid dominates in TAGs in traditional sunflower varieties and the levels of oleic and linoleic acid are linked to the FAD2 gene and thus unlikely to affect DGAT specificities (Pérez-Vich et al. 2002). In case of safflower, the higher specificity of DGAT towards oleic acids corroborate with our results indicating that DGAT is not the main enzyme contributing to TAG accumulation in this species. The specialisation towards 18:2 in *sn*-2 position of PC displayed by safflower PDAT, with about 3.5 times higher activity for 18:2 than 18:1, is another indication that PDAT may play a crucial role in the accumulation of the extremely linoleic-rich TAG (91 %) in this species. The direct transfer of newly synthesised 18:2 on PC (Stymne and Appelqvist 1978) to TAG by the very active PDAT will outcompete acylation of newly exported 18:1 from the plastid to DAG by DGAT. Instead this 18:1 will be acylated to the lysophosphatidylcholine formed by the PDAT reaction; a reaction catalysed by the very active lysophosphatidylcholine acyltransferase in safflower (Stymne and Stobart 1984). It is interesting to note that PDAT from sunflower and safflower, like PDAT from yeast and Arabidopsis (Dahlqvist et al. 2000; Ståhl et al. 2004) also have significant activity towards acyl groups at the *sn*-1 position of PC (about 25 % of the rate towards the *sn*-*2* position). Thus, it can be speculated that also position *sn*-*1* of PC plays a significant role in delivering acyl groups directly to TAG synthesis.

Previous work indicated that a diacylglycerol:diacylglycerol acyltransferase in developing castor and safflower seeds could also be involved in TAG synthesis (Stobart et al. 1997). These results were obtained before PDAT discovery (Banas et al. 2000; Dahlqvist et al. 2000) and could now be (at least partially) explained by PDAT enzyme activity. We have further shown that a soluble form of yeast PDAT has some DAG:DAG transacylation activity (Ghosal et al. 2007). In the presented study we were not able to detect any DAG:DAG acyltransferases in the membranes of the studied species, despite good PDAT activities, which cast some doubts of the significance of such reaction in TAG synthesis in these species.

In conclusion, the results presented indicate that the relative contribution of DGAT and PDAT in seed TAG biosynthesis and also their acyl specificities can be very different even in species belonging to the same family, like safflower and sunflower. Thus, it should be emphasised that although the enzymes involved in TAG synthesis in seeds from different species might be the same, the major flows of acyl groups through the different paths leading to TAG synthesis could be very different.

## Electronic supplementary material

Below is the link to the electronic supplementary material.

Supplementary material 1 (DOCX 72 kb)

## References

[CR1] Banas A, Dahlqvist A, Stahl U, Lenman M, Stymne S (2000). The involvement of phospholipid:diacylglycerol acyltransferases in triacylglycerol production. Biochem Soc Trans.

[CR2] Bates PD, Browse J (2012). The significance of different diacylgycerol synthesis pathways on plant oil composition and bioengineering. Front Plant Sci.

[CR3] Blight EG, Dyer WJ (1959). A rapid method of total lipid extraction and purification. Can J Med Sci.

[CR4] Bouvier-Nave P, Benvenise P, Oelkers P, Sturley S, Schaller H (2000). Expression in yeast and tobacco of plant cDNAs encoding acyl-CoA:diacylglycerol acyltransferase. Eur J Biochem.

[CR5] Cases S, Smith SJ, Zheng YW, Myers HM, Lear SR, Sande E, Novak S, Collins C, Welch CB, Luis AJ, Erickson SK, Farese RV (1998). Identification of a gene encoding an acyl-CoA: diacylglycerol acyltransferase, a key enzyme in triacylglycerol synthesis. Proc Natl Acad Sci USA.

[CR6] Chapman KD, Dyer JM, Mullen RT (2012). Biogenesis and functions of lipid droplets in plants: thematic review series: lipid droplet synthesis and metabolism: from yeast to man. J Lipid Res.

[CR7] Dahlqvist A, Ståhl U, Lenman M, Banas A, Lee M, Sandagar L, Ronne H, Stymne S (2000). Phospholipid:diacylglycerol acyltransferase: an enzyme that catalyzes the acyl-CoA-independent formation of triacylglycerol in yeast and plants. Proc Natl Acad Sci USA.

[CR8] Ghosal A, Banas A, Ståhl U, Dahlqvist A, Lindqvist Y, Stymne S (2007). *Saccharomyces cerevisiae* phospholipid:diacylglycerol acyl transferase (PDAT) devoid of its membrane anchor region is a soluble and active enzyme retaining its substrate specificities. Biochim Biophys Acta.

[CR9] Hernández ML, Whitehead L, He Z, Gazda V, Gilday A, Kozhevnikova E, Vaistij FE, Larson TR, Graham IA (2012). A cytosolic acyltransferase contributes to triacylglycerol synthesis in sucrose-rescued *Arabidopsis* seed oil catabolism mutants. Plant Physiol.

[CR10] Hobbs DH, Lu C, Hills MJ (1999). Cloning of a cDNA encoding diacylglycerol acyltransferase from *Arabidopsis thaliana* and its functional expression. FEBS Lett.

[CR11] Huang AHC (1992). Oil bodies and oleosins in seeds. Annu Rev Plant Physiol Plant Mol Biol.

[CR12] Kanda P, Wells MA (1986). Dihexanoyl phosphatidylethanolamine: effect of a head group charge on rates of alkaline and phospholipase-a2 catalyze dehydrolyzes. Chem Phys Lipids.

[CR13] Kennedy EP (1961). Biosynthesis of complex lipids. Fed Proc Fed Am Soc Exp Biol.

[CR14] Kroon JTM, Wei WX, Simon WJ, Slabas AR (2006). Identification and functional expression of type 2 acyl-CoA:diacylglycerol acyltransferase (DGAT2) in developing castor bean seeds which has high homology to the major triglyceride biosynthetic enzyme of fungi and animals. Phytochemistry.

[CR15] Lardizabal KD, Mai JT, Wagner NW, Wyrick A, Voelker T, Hawkins DJ (2001). DGAT2 is a new diacylglycerol acyltransferase gene family—purification, cloning, and expression in insect cells of two polypeptides from *Mortiella ramaniana* with diacylglycerol acyltransferases activity. J Biol Chem.

[CR16] Liu Q, Siloto RM, Lehner R, Stone SJ, Weselake RJ (2012). Acyl-CoA:diacylglycerol acyltransferase: molecular biology, biochemistry and biotechnology. Prog Lipid Res.

[CR17] Lu C, Xin Z, Ren Z, Miquel M, Browse J (2009). An enzyme regulating triacylglycerol composition is encoded by the ROD1 gene of *Arabidopsis*. Proc Natl Acad Sci USA.

[CR18] Mhaske V, Beldjilali K, Ohlrogge J, Pollard M (2005). Isolation and characterization of an *Arabidopsis thaliana* knockout line for phospholipid:diacylglycerol transacylase gene (At5g13640). Plant Physiol Biochem.

[CR19] Murphy DJ (2005). Plant lipids: biology, utilization and manipulation.

[CR20] Oelkers P, Tinkelenberg A, Erdeniz N, Cromley D, Billheimer JT, Sturley SL (2000). A lecithin cholesterol acyltransferase-like gene mediates diacylglycerol esterification in yeast. J Biol Chem.

[CR21] Ohlrogge JB, Browse J (1995). Lipid biosynthesis. Plant Cell.

[CR22] Pérez-Vich B, Fernándes-Martinez JM, Grodona M, Knapp SJ, Berry ST (2002). Stearoyl-ACP and oleoyl-PC desaturase genes cosegregate with quantitative trait loci underlying high stearic and high oleic acid mutant phenotypes in sunflower. Theor Appl Genet.

[CR23] Saha S, Enugutti B, Rajakumari S, Rajasekharan R (2006). Cytosolic triacylglycerol biosynthetic pathway in oilseeds: molecular cloning and expression of peanut cytosolic diacylglycerol acyltransferase. Plant Physiol.

[CR24] Shockey JM, Gidda SK, Chapital DC, Kuan JC, Dhanoa PK, Bland JM, Rothstein SJ, Mullen RT, Dyer JM (2006). Tung tree GDAT1 and DGAT2 have non redundant functions in triacylglycerol biosynthesis and are localized to different subdomains of the endoplasmic reticulum. Plant Cell.

[CR25] Somerville C, Browse J, Jaworski JG, Ohlrogge JB, Buchanan BB, Gruissem W, Jones JL (2001). Lipids. Biochemistry and molecular biology of plants.

[CR26] Sörensen BM, Furukawa-Stoffer TL, Marshall KS, Page EK, Mir Z, Forster RJ, Weselake RJ (2005). Storage lipid accumulation and acyltransferase action in developing flax seeds. Lipids.

[CR27] Ståhl U, Carlsson A, Lenman M, Dahlqvist A, Huang B, Banas W, Banas A, Stymne S (2004). Cloning and functional characterization of a phospholipid:diacylglycerol acyltransferase from *Arabidopsis*. Plant Physiol.

[CR28] Stobart AK, Stymne S (1985). The regulation of fatty acid composition of the triacylglycerols in microsomal preparations from avocado mesocarp and developing cotyledons of safflower. Planta.

[CR29] Stobart AK, Mancha M, Lenman M, Dahlqvist A, Stymne S (1997). Triacylglycerols are synthesised and utilized by transacylation reactions in microsomal preparations of developing safflower (*Carthamus tinctorius* L.) seeds. Planta.

[CR30] Stymne S, Appelqvist LA (1978). The biosynthesis of linoleate from oleoyl-CoA via oleoyl-phosphatidylcholine in microsomes of developing safflower seeds. Eur J Biochem.

[CR31] Stymne S, Stobart AK (1984). Evidence for the reversibility of the acyl-CoA:lysophosphatidylcholine acyltransferase in microsomal preparations from developing safflower (*Carthamus tinctorius* L.) cotyledons and rat liver. Biochem J.

[CR32] Stymne S, Stobart AK, Stumpf PK (1987). Triacylglycerol biosynthesis. The biochemistry of plants.

[CR33] Triki S, Ben Hamida J, Mazliak P (2000). Diacylglycerol acyltransferase in maturing sunflower seeds. Biochem Soc Trans.

[CR34] Vogel G, Browse J (1996). Cholinephosphotransferase and diacylglycerol acyltransferase. Plant Physiol.

[CR35] Weselake RJ, Pomeroy MK, Furukawa TL, Golden JL, Little DB, Laroche A (1993). Developmental profile of diacylglycerol acyltransferase in maturing seeds of oilseed rape and safflower and in microspore derived cultures of oil rape. Plant Physiol.

[CR36] Wiberg E, Tillberg E, Stymne S (1994). Substrates of diacylglycerol acyltransferase in microsomes from developing seeds. Phytochemistry.

[CR37] Zhang M, Fan J, Taylor D, Ohlrogge J (2009). DGAT1 and PDAT1 acyltransferases have overlapping functions in Arabidopsis triacylglycerol biosynthesis and are essential for normal pollen and seed development. Plant Cell.

[CR38] Zou J, Wei Y, Jako C, Kumar A, Selvaraj G, Taylor DC (1999). The Arabidopsis thaliana TAG1 mutant has a mutation in a diacylglycerol acyltransferase gene. Plant J.

