# Supplementary material for: Activities of acyl-CoA:diacylglycerol acyltransferase (DGAT) and phospholipid:diacylglycerol acyltransferase (PDAT) in microsomal preparations of developing sunflower and safflower seeds
Source: Planta. 2013 Mar 29;237(6):1627–36. doi: 10.1007/s00425-013-1870-8 (PMC3664747; doi:10.1007/s00425-013-1870-8)
Supplement: Supplementary file 1 — Supplementary material 1 (DOCX 72 kb) [file 425_2013_1870_MOESM1_ESM.docx]

**Supplementary materials**

Activities of acyl-CoA:diacylglycerolacyltransferase (DGAT) and phospholipid:diacylglycerolacyltransferase (PDAT) in microsomal preparations of developing sunflower and safflower seeds

**Planta**

Walentyna Banaś^1*^, Alicia Sanchez Garcia^2^, Antoni Banaś^3^, Sten Stymne^4^

^1^Institute of Biology, University of Natural Sciences and Humanities, Prusa 12,
08 -110 Siedlce, Poland

^2^Instituto de la Grasa, CSIC, Sevilla, Spain

^3^Intercollegiate Faculty of Biotechnology of University of Gdansk and Medical University of Gdansk, Kladki 24, 80-822 Gdansk, Poland

^4^ Department of Plant Breeding and Biotechnology, SLU, Alnarp, Sweden

* Corresponding author. *E-mail address*: [wbanas@uph.edu.pl](mailto:wbanas@uph.edu.pl) (W. Banaś).

Table S1. Time dependency of TAG synthesis catalysed by microsomal sunflower PDAT and DGAT.

| **Substrates added** | **nmol [^14^C]TAG formed / mg microsomal protein during incubation time** | | | |
| --- | --- | --- | --- | --- |
|  | **5 min** | **15min** | **30 min** | **60 min** |
| **[^14^C]18:1-DAG + 18:1-CoA**  (PDAT + DGAT activity) | 7,8±0,5 | 16,7±0,4 | 21,8±3,5 | 28,8±3,4 |
| **[^14^C]18:1-DAG** (PDAT activity) | 0,9±0,1 | 1,8±0,3 | 3,2±0,7 | 4,4±0,2 |

Means ± S.D. shown (n≥3). Microsomal membranes used in the assays were prepared from sunflower seeds at middle-late stage of development (30 days after flowering). Incubation conditions were as described in Material and Methods section.

Table S2. Time dependency of the reaction rate of TAG synthesis catalysed by microsomal sunflower DGAT.

| **Substrates added** | **nmol di-6:0-[^14^C]TAG formed / mg microsomal protein during incubation time** | | | |
| --- | --- | --- | --- | --- |
|  | **5 min** | **15min** | **30 min** | **60 min** |
| **di-6:0-DAG + [^14^C]18:1-CoA**  (DGAT activity) | 11,1±1,2 | 19,1±0,5 | 25.8±2,3 | 33,7±5,2 |

Means ± S.D. shown (n≥3). Microsomal membranes used in the assays were prepared from sunflower seeds at middle-late stage of development (30 days after flowering). Incubation conditions were as described in Material and Methods section.

Table S3. Time dependency of TAG synthesis catalysed by microsomal safflower PDAT and DGAT.

| **Substrates added** | **nmol [^14^C]TAG formed / mg microsomal protein during incubation time** | |
| --- | --- | --- |
|  | **5 min** | **15min** |
| **[^14^C]18:1-DAG + 18:1-CoA**  (PDAT + DGAT activity) | 6,7±0,4 | 14,2±0,4 |
| **[^14^C]18:1-DAG** (PDAT activity) | 4,1±0,7 | 7,7±0,2 |

Means ± S.D. shown (n≥3). Microsomal membranes used in the assays were prepared from safflower seeds at middle stage of development (17 days after pollination). Incubation conditions were as described in Material and Methods section.

Table S4. Time dependency of TAG synthesis catalysed by microsomal safflower DGAT.

| **Substrates added** | **nmol di-6:0-[^14^C]TAG formed / mg microsomal protein during incubation time** | |
| --- | --- | --- |
|  | **5 min** | **15min** |
| **di-6:0-DAG + [^14^C]18:1-CoA**  (DGAT activity) | 6,2±0,4 | 11,2±0,2 |

Means ± S.D. shown (n≥3). Microsomal membranes used in the assays were prepared from safflower seeds at middle stage of development (17 days after pollination). Incubation conditions were as described in Material and Methods section.

Table S5. TAG synthesis from different combinations of substrates by microsomal fractions from developing sunflower seeds.

| **Substrates added** | **nmol [^14^C]TAG formed / min x mg protein** | | | | |
| --- | --- | --- | --- | --- | --- |
|  | **15 DAF** | **20 DAF** | **25 DAF** | **30 DAF** | **40 DAF** |
| **[^14^C]18:1DAG** (PDAT activity –only exogenous DAG) | **0.19**±0.05 | **0.25**±0.06 | **0.22**±0.04 | **0.22**±0.04 | **0.22**±0.05 |
| **[^14^C]18:1-DAG + 18:1-CoA** (PDAT + DGAT activity – only exogenous DAG) | 1.72±0.22 | 1.78±0.28 | 1.59±0.24 | 1.37±0.22 | 1.34±0.08 |
| **Calculated DGAT activity** (use for PDAT/DGAT ratio - only exogenous DAG) | **1.53** | **1.53** | **1.37** | **1.15** | **1.12** |
| **[^14^C]18:1-CoA** (DGAT activity – endogenous DAG) | 1.23±0,03 | 1.45±0,03 | 0.75±0,08 | 0.50±0,05 | 0,26±0,08 |
|  |  |  |  |  |  |
| **[^14^C]18:1DAG + 18:2-CoA** (PDAT + DGAT activity – only exogenous DAG) | 2.93±0.17 | 4.16±0.28 | 3.90±0.26 | 3.58±0.17 | 2.32±0.06 |
| **Calculated DGAT activity** (use for PDAT/DGAT ratio - only exogenous DAG) | **2.74** | **3.91** | **3.68** | **3.36** | **2.10** |
| **[^14^C]18:2-CoA** (DGAT activity – endogenous DAG) | 0,73±0,13 | 1,02±0,08 | 0,57±0,07 | 0,48±0,04 | 0,24±0,05 |
|  |  |  |  |  |  |
| **di-6:0-DAG + [^14^C]18:1-CoA**  (DGAT activity) | 2.82±0.86 | 3.00±0.21 | 1.88±0.12 | 1.82±0.04 | 1.79±0.19 |
| **di-6:0-DAG + [^14^C]18:2-CoA**  (DAGAT activity) | 3.18±0.12 | 6.48±0.18 | 3.97±0.14 | 3.80±0.43 | 3.22±0.08 |

In experiments with di-6:0-DAG + [^14^C]FA-CoA only synthesised di-6:0-TAG is presented; the amount of [^14^C]TAG synthesised from endogenous DAG and [^14^C]FA-CoA was low (from 0.7% to 4,8% of synthesised di-6:0-TAG value).

Means ± S.D. shown (n≥3); DAF = seeds days after flowering used for microsomal membrane preparations

Table S6. TAG synthesis from different combinations of substrates by microsomal fractions from developing safflower seeds.

| **Substrates added** | **nmol [^14^C]TAG formed / min x mg protein** | | | |
| --- | --- | --- | --- | --- |
|  | **12 DAP** | **17 DAP** | **22 DAP** | **27 DAP** |
| **[^14^C]18:1-DAG** (PDAT activity –only exogenous DAG) | **0.57**±0.08 | **0.66**±0.09 | **0.68**±0.04 | **0.36**±0.02 |
| **[^14^C]18:1-DAG + 18:1-CoA** (PDAT + DGAT activity – only exogenous DAG) | 1.56±0.27 | 1.16±0.04 | 1.16±0.12 | 0.60±0.03 |
| **Calculated DGAT activity** (use for PDAT/DGAT ratio - only exogenous DAG) | **0.99** | **0.50** | **0.48** | **0.24** |
| **[^14^C]18:1-CoA** (DGAT activity – endogenous DAG) | 0.57±0,02 | 0.38±0,03 | 0.24±0,02 | 0.14±0,02 |
|  |  |  |  |  |
| **[^14^C]-18:1DAG + 18:2-CoA** (PDAT + DGAT activity – only exogenous DAG) | 0.99±0.08 | 0.92±0.09 | 0.89±0.04 | 0.51±0.08 |
| **Calculated DGAT activity** (use for PDAT/DGAT ratio - only exogenous DAG) | **0.42** | **0.26** | **0.21** | **0.15** |
| **[^14^C]18:2-CoA** (DGAT activity – endogenous DAG) | 0,37±0,01 | 0,26±0,03 | 0,19±0,01 | 0,08±0,01 |
|  |  |  |  |  |
| **di-6:0-DAG + [^14^C]18:1-CoA**  (DGAT activity) | 3.28±0.26 | 2.57±0.08 | 2.10±0.17 | 1.67±0.06 |
| **di-6:0-DAG + [^14^C]18:2-CoA**  (DGAT activity) | 2.02±0.13 | 1.98±0.05 | 1.69±0.06 | 0.95±0.1 |

In experiments with di-6:0-DAG + [^14^C]FA-CoA only synthesised di-6:0-TAG is presented; the amount of [^14^C]TAG synthesised from endogenous DAG and [^14^C]FA-CoA was low (from 2.3% to 14,3% of synthesised di-6:0-TAG value).

Means ± S.D. shown (n≥3); DAP = seeds days after pollination used for microsomal membrane preparations


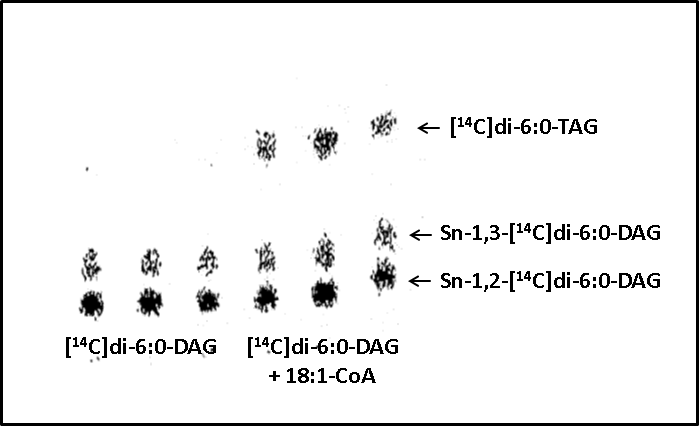


Fig. S1. Autoradiogram of TLC plate with separated lipids from incubations of microsomes from developing safflower seeds (triplicate samples) with radioactive di-6:0-DAG with and without addition of unlabelled 18:1-CoA. Incubation conditions identical to that described in Materials and Methods except that radioactive di-6:0-DAG and non-radioactive acyl-CoA were used as substrates. Only traces of radioactivity were seen in triacylglycerols without addition of acyl-CoA indicating that di-6:0-DAG is, at the most, a poor acceptor and that the microsomes contained too low endogenous acyl-CoA concentration for allowing detection of significant DGAT activity.
